# Multimodal microscopic and spectroscopic characterisation of heterogeneous iron oxyhydroxides in the human globus pallidus

**DOI:** 10.1007/s10534-026-00860-4

**Published:** 2026-07-23

**Authors:** Martin Kopáni, Daniel Kosnáč, Pavol Gemeiner, Jana Hlinková, Pavol Janega, Ján Pánik, Štefan Polák

**Affiliations:** 1 Institute of Medical Physics and Biophysics, Faculty of Medicine, Comenius University, Sasinkova 2, 811 08 Bratislava, Slovakia; 2 Department of Graphic Arts Technology and Applied Photochemistry, Institute of Polymer and Synthetic Materials, Faculty of Chemical and Food Technology, Slovak University of Technology, Radlinského 9, 812 37 Bratislava, Slovakia; 3 Institute of Clinical Dentistry, Faculty of Dentistry, University of Oslo, Pb 1109, Blindern, 0317 Oslo, Norway; 4 Institute of Pathological Anatomy, Faculty of Medicine, Comenius University, Sasinkova 4, 811 08 Bratislava, Slovakia; 5 Institute of Anatomy, Faculty of Medicine, Comenius University, Sasinkova 2, 811 08 Bratislava, Slovakia

**Keywords:** Raman spectroscopy, Globus pallidus, SEM–EDX, Neurodegeneration, Ferritin, Magnetite, Iron oxides

## Abstract

Iron is essential for neuronal metabolism, neurotransmitter synthesis, and enzymatic function; however, dysregulated accumulation contributes to oxidative stress and neurodegeneration. The basal ganglia, particularly the *globus pallidus*, represent a hotspot for iron deposition, yet the precise structural forms and their implications remain incompletely understood. Here, a multimodal approach was applied combining Raman microspectroscopy, light microscopy, transmission (TEM) and scanning electron microscopy coupled with energy-dispersive X-ray analysis (SEM–EDX) to characterise iron-rich deposits in post-mortem human *globus pallidus*. Tissue samples from six individuals without neurological disease were examined. Perls’ staining revealed iron-positive, spherical inclusions 10–20 µm in diameter. Raman spectroscopy revealed bands at 268–278, 490, 526, and 603 cm^−1^, as well as broader signals at 1259–1349 cm^−1^, consistent with magnetite, maghemite, hematite, and ferritin-like structures. Additional vibrations in the 682–1532 cm^−1^ range indicated interactions with organic matrices, such as protein or lipid components. SEM–EDX identified both regular and irregular iron-rich particles with multielemental composition, including C, O, Al, Si, P, S, Ca, Cr, and Ni, in addition to Fe. TEM examination showed the micrometre-sized particles of hematite and aggregation of ferrihydrite. These findings suggest that iron deposits in the *globus pallidus* comprise heterogeneous mixtures of oxides and hydroxides with variable crystallinity. Depending on their crystallinity and surface reactivity, such phases may represent a potential pool of redox-active iron; however, the present study did not assess markers of oxidative stress, and their physiological versus pathological significance remains to be established.

## Introduction

Iron is an essential trace element in the central nervous system (CNS), playing a pivotal role in fundamental cellular processes, including mitochondrial respiration, myelin synthesis, neurotransmitter synthesis, and the function of enzymes dependent on redox reactions (Quintana and Gutiérrez 2010; Rouault 2013; Ward et al. 2014). Iron accumulation is thought to be related to the high metabolic activity of the region, age, its abundance of oligodendrocytes and myelinated fibres. Iron is unevenly distributed within the human brain, with the highest concentrations reported in the *globus pallidus* (Zecca et al. 2004), whilst others have reported higher level in putamen (Ramos et al. 2014).

Iron accumulation is critical, as excess free iron drives the formation of reactive oxygen species (ROS) through Fenton chemistry, leading to lipid peroxidation, protein oxidation, and DNA damage (Cornell and Schwertmann 2003; Dixon and Stockwell 2014). Excessive iron deposition may also result from disturbances in brain iron homeostasis. Dysregulation of proteins involved in iron transport, storage, and export, including transferrin, ferritin, ferroportin, and ceruloplasmin, can lead to abnormal iron accumulation within neurons and glial cells (Rouault 2013; Ward et al. 2014). It is closely linked to neuroinflammatory processes, which can promote iron retention, while excess iron can in turn amplify inflammatory responses, creating a cycle that may contribute to neurodegeneration. Conversely, inflammation-driven sequestration of iron as ferritin within glia and microglia may reduce iron availability to neurons, potentially contributing to a localised, functional neuronal iron deficiency. Neuroinflammation is associated with the pathogenesis of several major neurodegenerative disorders such as Parkinson’s disease, Huntington’s disease, and Alzheimer’s disease (Ke and Qian 2003; Thomas and Jankovic 2004; Castellani et al. 2012). Numerous studies have demonstrated a significant age-related rise in total brain iron content, particularly in deep grey matter structures such as the substantia nigra, *globus pallidus*, and putamen. This gradual increase reflects lifelong iron deposition and altered iron homeostasis within neural tissue, and is considered a normal feature of brain ageing rather than a pathological process per se, although it may contribute to vulnerability under neurodegenerative conditions (Zecca et al. 2004; Hagemeier et al. 2012; Ward et al. 2014).

Several complementary analytical techniques have been used to identify and characterise iron-containing phases in the human brain. Histochemical approaches, particularly Perls’ Prussian blue staining, are routinely applied to visualise ferric iron distribution, but they do not provide phase-specific chemical identification. Higher-resolution insights have been obtained using electron microscopy (TEM/SEM), often combined with energy-dispersive X-ray spectroscopy (EDS), which enables detection of nanoscale iron-rich particles and confirmation of elemental composition, although without definitive crystallographic discrimination (Guo et al. 2026).

Phase identification has been further refined using selected area electron diffraction (SAED), which allows distinction between amorphous ferritin-like iron phases and crystalline iron oxides. Synchrotron-based techniques, especially X-ray absorption spectroscopy (XAS; XANES/EXAFS), have consistently shown that the dominant form of iron in the human brain is ferritin-like, with a ferrihydrite-like mineral core and Fe^3+^-dominated coordination environment (Willans et al. 2025). Synchrotron X-ray fluorescence (XRF) imaging has further demonstrated heterogeneous iron distribution across brain regions, particularly in iron-rich structures such as the basal ganglia, putamen and substantia nigra. Magnetic measurements, including SQUID magnetometry, together with Raman and Mössbauer spectroscopy, have provided evidence that trace amounts of ferrimagnetic iron oxides, most notably magnetite and maghemite, may be present in human brain tissue. However, their abundance, spatial distribution, and biological versus environmental origin remain unresolved and are subject to ongoing debate (Svobodová et al. 2019).

Current knowledge supports the view that brain iron is predominantly stored in ferritin-like nanostructures (5Fe_2_O_3_·9H_2_O), which serve as a safe iron reservoir and limit redox activity (Zecca et al. 2004). Nevertheless, several key aspects remain uncertain. In particular, the existence, prevalence, and physiological relevance of crystalline iron oxides such as hematite (α-Fe_2_O_3_), maghemite (γ-Fe_2_O_3_) or magnetite (Fe_3_O_4_) in the healthy human brain are not conclusively established. It also remains unclear whether detected magnetic iron phases represent biologically controlled biomineralisation products, pathological by-products of iron dysregulation, or external particulate contaminants. Furthermore, the exact nanoscale structural relationship between ferritin cores and potential mineral-like iron oxide phases is still not fully resolved, largely due to limitations in spatial resolution and phase sensitivity of current in situ techniques (Cowley et al. 2000; Collingwood and Telling 2016; Koralewski et al. 2018). While there is strong consensus that ferritin-like iron dominates brain iron storage, the presence, origin, and functional significance of crystalline iron oxide nanoparticles remain open questions requiring further multimodal and correlative investigations.

Understanding the role of iron in neurodegenerative mechanisms requires the precise chemical identification and localisation of these iron deposits. Traditionally, conventional histochemical methods such as Perls’ Prussian blue have been employed to visualise ferric iron (Fe^3+^) deposits. While useful for determining the spatial distribution of iron, these techniques provide limited information about its specific mineral structure or chemical state (Meguro et al. 2007). Similarly, non-invasive techniques like magnetic resonance imaging (MRI) can quantify iron levels in vivo but lack the resolution and chemical specificity to distinguish between different iron oxide mineral phases (Langkammer et al. 2010). Conversely, Raman spectroscopy is a powerful, non-destructive analytical technique based on the inelastic scattering of monochromatic light, which provides a unique vibrational fingerprint of a sample’s molecular composition (Matousek and Stone 2016). It enables the detailed identification of crystalline and nanocrystalline phases of iron oxides and hydroxides at the microscale, allowing for the distinction between them (de Faria et al. 1997; Slavov et al. 2010). When combined with scanning electron microscopy (SEM) for high-resolution morphological imaging and energy-dispersive X-ray microanalysis (EDX) for elemental composition, this multimodal approach provides a comprehensive characterisation of mineral deposits within a biological context (Goldstein 2003; Hussain et al. 2023).

This study provides a correlative, multimodal characterisation of iron-rich deposits in the human *globus pallidus*, spanning from nanoscale ferrihydrite aggregates to well-formed microscale crystals, and reveals a complex mixture of crystalline and amorphous iron (oxyhydr)oxide phases embedded in a multielemental organic matrix, with potential implications for the intrinsic redox activity of the healthy human brain.

The objective of this study is not to show any markers of oxidative stress in connection with iron accumulation, but to characterise the chemical composition and identify the specific forms of iron present in deposits within the human *globus pallidus* using a correlative spectroscopic and microscopic approach. We hypothesise that these deposits do not consist of a single, uniform iron species but are rather heterogeneous mixtures of different iron oxides embedded within an organic matrix, reflecting complex biomineralisation processes. Elucidating the precise nature of these deposits is a critical step towards understanding their pathological consequences in neurodegenerative diseases.

## Materials and methods

### Samples

Human *post-mortem* brain tissue from the *globus pallidus externus* was obtained during autopsies at the Department of Pathology, Comenius University, Bratislava, Slovakia. In accordance with relevant legislation (§48, Section 3 of Act No. 581/2004 Coll.), and based on the order of the Health Care Surveillance Authority of the Slovak Republic, autopsies were performed, which included indicated laboratory examinations. The analyses presented in this publication were performed as part of these autopsies–related laboratory tests. The Ethics Committee of the Faculty of Medicine, Comenius University, confirmed that no additional ethical approval is required due to the regulatory oversight by the Health Care Surveillance Authority of the Slovak Republic. Furthermore, the Health Care Surveillance Authority of the Slovak Republic agrees to the publication of the anonymised results, which do not contain any personal data that could lead to patient identification (6521/2026/960; 22nd January 2026). Samples were collected from six individuals (three males, three females, age 19–83 years) with no clinical evidence of neurological or movement disorders (Table 1). Causes of death were unrelated to neurological disease. All procedures were conducted in compliance with the Declaration of Helsinki.

**Table 1:** Clinical characteristics of sampled individuals

Sample	Gender	Age (years)	Cause of death	Post-mortem interval (hours)	Occupation
S1	M	53	Cirrhosis	7	Teacher
S2	F	69	Heart failure	10	Lawyer
S3	F	83	Heart failure	8	Saleswoman
S4	M	19	Nephritis	6	Student
S5	M	67	Heart failure	9	Builder
S6	F	72	Thrombosis	10	Cook

### Light microscopy

Samples were fixed in 10% formaldehyde for 24 h, embedded in paraffin blocks, and subsequently cut by microtome into 5 µm-thin sections. The sections were mounted on gelatin-coated slides for light microscopy. For general morphological and iron localisation purposes, sections were stained using Perls’ method, which detects Fe(III) ions, and then covered by a cover glass for examination under a light microscope.

### Raman spectroscopy

After initial examination with a light microscope, the cover glass was carefully removed from the slides designated for spectroscopic analysis. Raman spectra were acquired using a DXRi 3xi Raman imaging microscope (Thermo Scientific™, USA) equipped with a 633 nm excitation laser operating at a power of 7.0 mW. Spectra were collected with an exposure time of 0.5 s per accumulation. Raman spectra were obtained in the high-resolution range of 50–1800 cm^−1^. The assignment of observed Raman shifts to specific iron oxide phases and organic compounds was based on a comprehensive review, as summarised in Tables 2 and 3.

**Table 2:** Raman shifts for iron oxide phases reported in the literature and used for phase assignments

Iron oxide	Raman shift (cm^−1^)	Literature
Magnetite	193 (weak), 298, 301.6, 306 (weak), 319, 418, 513, 533, 538 (weak), 550, 662.7, 668 (strong), 676, 1332	(Dünnwald and Otto 1989; de Faria et al. 1997; Shebanova and Lazor 2003; Slavov et al. 2010)
Biologically induced magnetite^*^	700	(Perez-Gonzalez et al. 2010)
Hematite	221, 225 (strong), 247 (weak), 285, 293, 299 (strong), 397, 412 (strong), 498 (weak), 602, 613 (medium), 645	(de Faria et al. 1997; Slavov et al. 2010; Kim et al. 2022)
Maghemite	350 (strong), 500 (strong), 700 (strong), 1320, 1560	(de Faria et al. 1997; Slavov et al. 2010)
Goethite	243, 299, 385, 479, 550, 685, 993, 1160, 1314	(de Faria et al. 1997)
Lepidocrocite	245, 373, 493 (shoulder), 522, 650 (shoulder), 719, 1120, 1303	(de Faria et al. 1997)
2-line ferrihydrite	370, 510, 710, 1340	(Mazzetti and Thistlethwaite 2002)

^*^Biologically induced magnetite is magnetite formed as a result of biological activity within cells and tissues. Differences between biologically induced and abiotic magnetite are primarily related to crystal size, crystallinity, lattice defects, internal strain, and oxidation state. These structural variations can affect the Raman spectrum, resulting in shifts in peak positions, peak broadening, and changes in band intensities

**Table 3:** Raman shifts for organic compounds reported in the literature and used for assignments

Organic compound	Raman shift (cm^−1^)	Literature
Amide I (protein)	1640–1730	Spencer et al. (2021)
Amide II (protein)	1440–1560	Spencer et al. (2021)
Amide III (protein)	1200–1300	Spencer et al. (2021)
PO_4_^3−^ group	591, ~ 965	Shahabi et al. (2016)
CH_2_/CH_3_ deformation (lipid/protein)	~ 1290–1460	Hartmann et al. (2020); Spencer et al. (2021)
C–C stretch (protein backbone)	900–980	Rygula et al. (2013)
C–COO group	~ 605	Spencer et al. (2021)
Porphyrins, heme groups	1500–1600	Huang et al. (2004)
Melanin, lipofuscin	~ 1340–1460	Huang et al. (2004)

### Scanning electron microscopy and energy-dispersive x-ray spectroscopy (SEM–EDX)

After Raman analysis, the tissue sections were coated with a 20 nm layer of carbon and examined using a ZEISS EVO LS 15 scanning electron microscope (SEM) operated at an accelerating voltage of 15 kV. Elemental composition was determined using an AMETEK EDAX EDS silicon drift detector. Spectra were acquired for a period of 200 s over an energy range of 0.16–10 keV.

### Transmission electron microscopy and electron diffraction (TEM/SAED)

Non-contrasted ultrathin sections were mounted on nickel grids and investigated using a JEOL 840B transmission electron microscope (TEM) with an acceleration voltage of 150 kV. To determine the iron oxide phase, energy-dispersive X-ray spectroscopy (EDX) was performed using a KEVEX 3205–1200 detector to confirm elemental composition. The selected area electron diffraction (SAED) method was used to determine the crystalline structure. The International Centre for Diffraction Data (ICDD) database was used for phase identification, specifically ICDD card no. 33–0664 for hematite and no. 29–0712 for ferrihydrite.

## Results

### Histochemistry

**Fig. 1:**
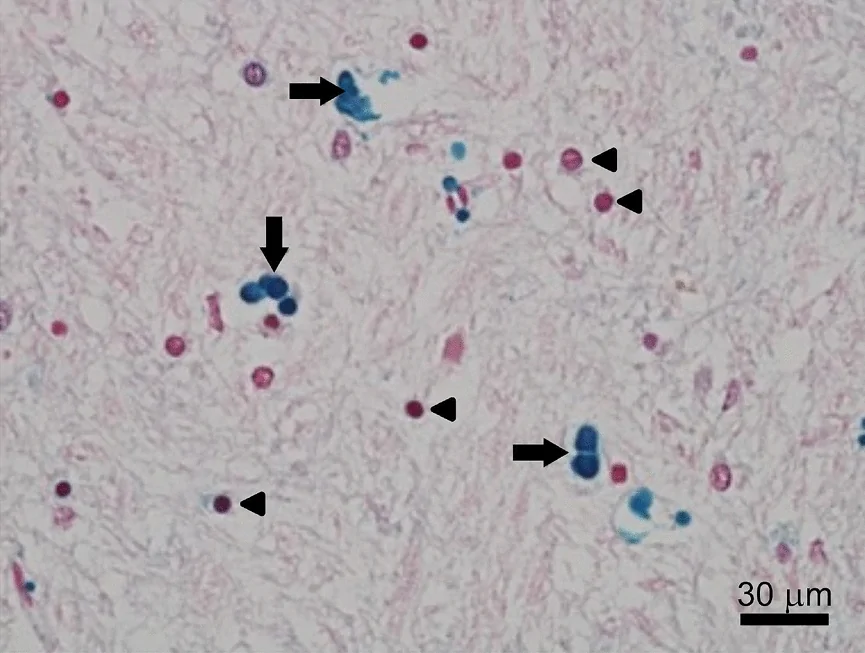
Light microscopy image of a human globus pallidus section (S1). The population of glial cells (arrowheads) with blue-stained deposits (black arrows) indicate the presence of Fe(III) ions revealed by Perls’ histochemical reaction. The deposits are located in the vicinity of glial cells

In the *globus pallidus*, neurons are large (35–70 µm), while glial cells are smaller (8–15 µm). Iron mainly accumulates in glial cells. Perls’ staining revealed spherical, blue-stained iron deposits (10–20 µm) localised near glial cells in the *globus pallidus* (Fig. 1). These deposits, which react positively for the presence of Fe(III) ions, were observed consistently across the examined samples. In these neurologically healthy tissues, iron storage is expected to be predominantly intracellular (mainly as ferritin within glia). However, given the range of crystalline iron oxides detected, an extracellular component cannot be excluded.

### Raman spectroscopy

**Fig. 2:**
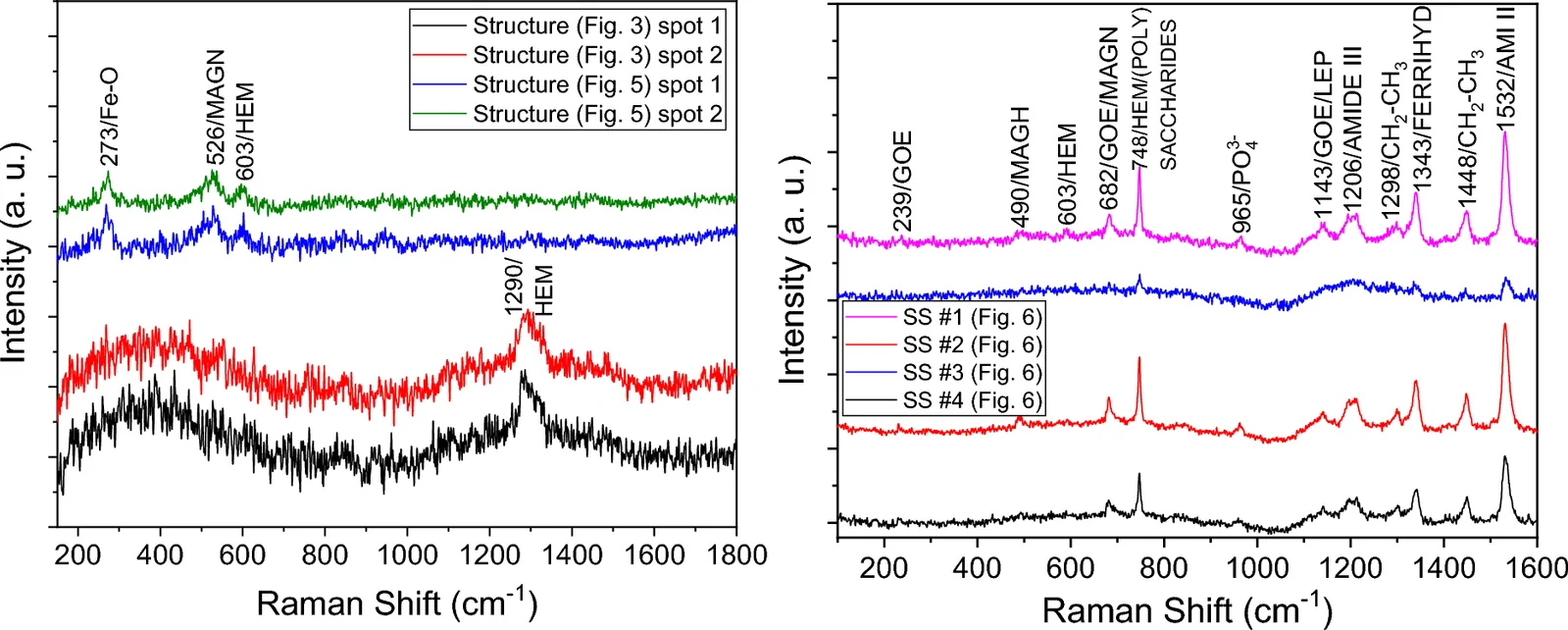
(Left) Raman spectra of the irregular iron-rich deposits collected from the structures in Fig. 3 and 5 (S1 and S3). (Right) Raman spectra collected from spherical structures (SS) in Fig. 6 (S6). Spectra were vertically shifted for clarity

Spectra revealed both less intense peaks (Fig. 2 left) and sharper peaks at higher wavenumbers (Fig. 2 right). Raman analysis of the iron deposits revealed several characteristic bands. Key vibrations corresponding to iron oxides were identified in the low-frequency region at 268–278 cm^−1^, along with distinct peaks at approximately 490 cm^−1^, 526 cm^−1^, and 603 cm^−1^. A broad spectral feature was consistently observed in the 1259–1349 cm^−1^ range. Additionally, a series of sharper, more intense peaks were detected at 682, 748, 965, 1143, 1206, 1298, 1343, 1448, and 1532 cm^−1^(Fig. 2). Based on literature assignments (Table 2), these spectral features indicate the presence of various iron oxyhydroxides and organic molecules such as proteins, lipids and saccharides.

### SEM–EDX

**Fig. 3:**
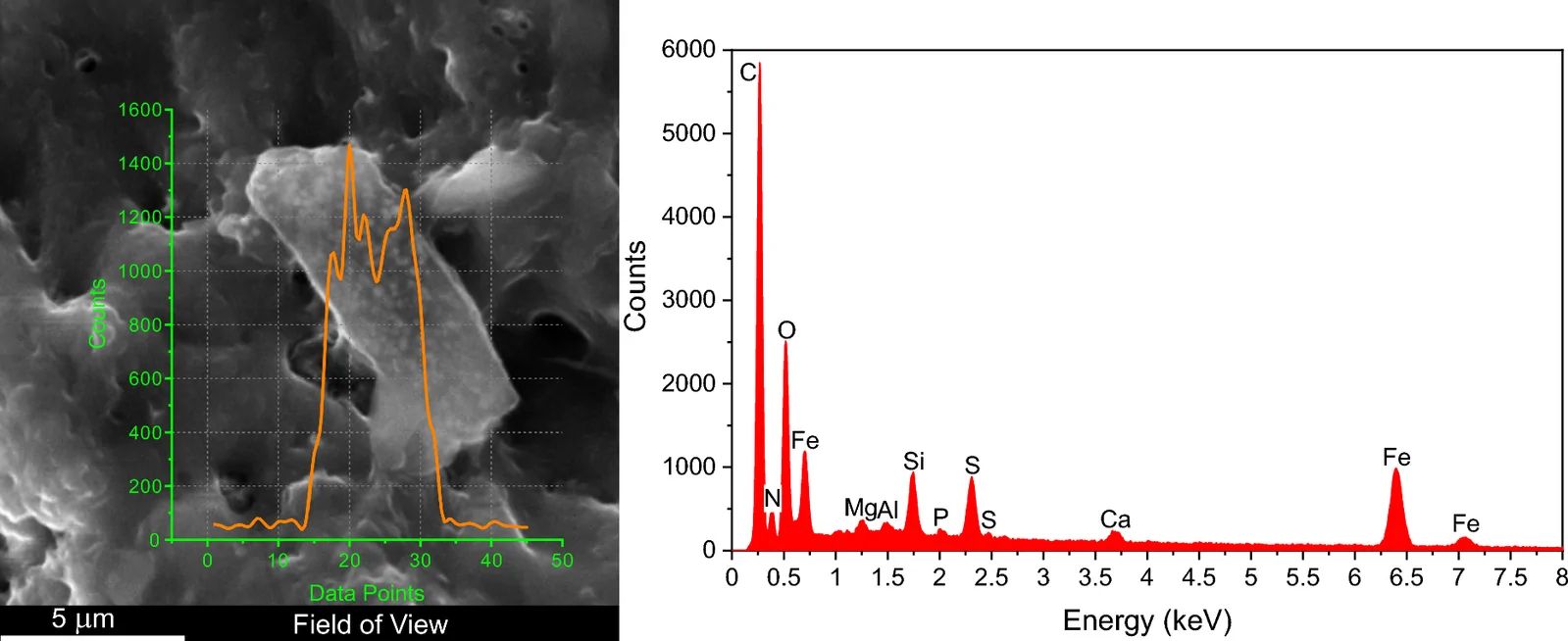
(Left) SEM image of a rectangular, iron-rich particle (S1), with an overlaid EDX line scan showing iron distribution. (Right) The corresponding EDX spectrum reveals the presence of C, N, O, Mg, Al, Si, P, S, Ca, and Fe

**Fig. 4:**
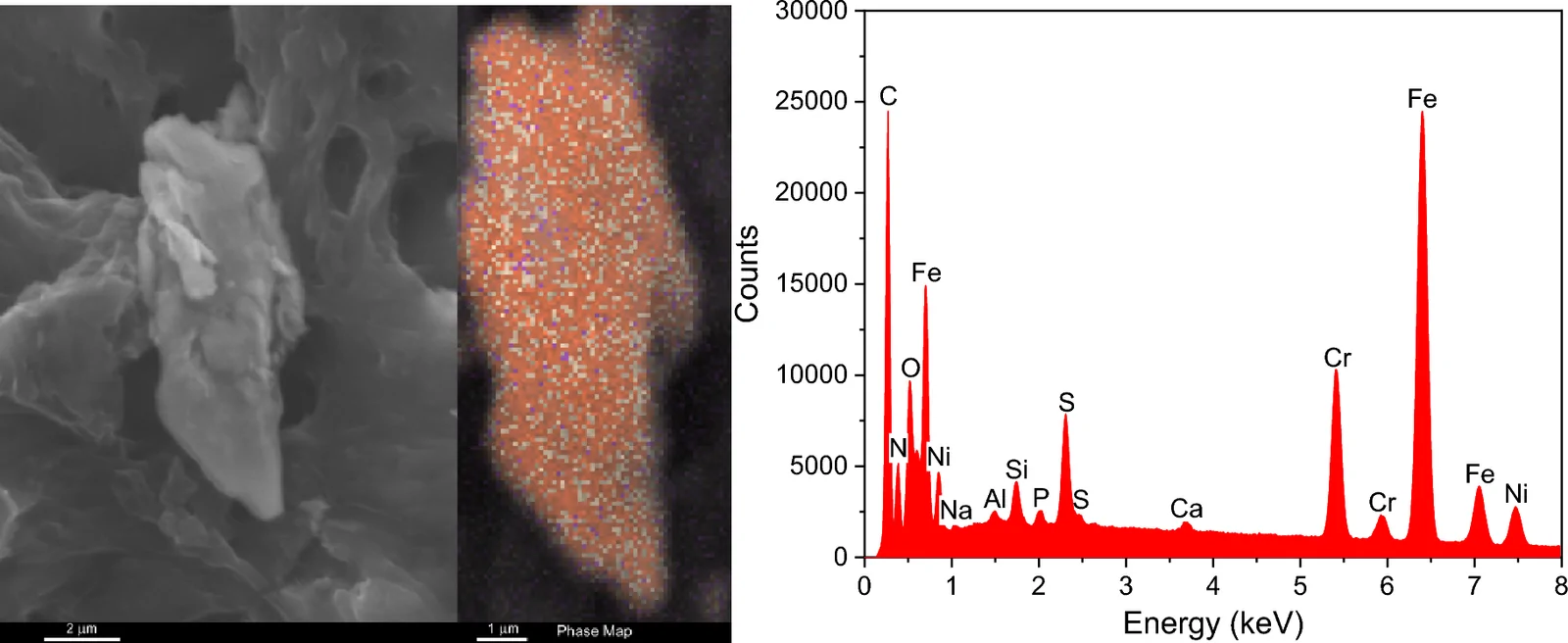
(Left) SEM image of an irregular iron-rich structure (S2) and elemental phase map highlighting the distribution of iron, with the corresponding EDX spectrum showing peaks for C, N, O, Na, Al, Si, P, S, Ca, Cr, Fe, and Ni (Right)

**Fig. 5:**
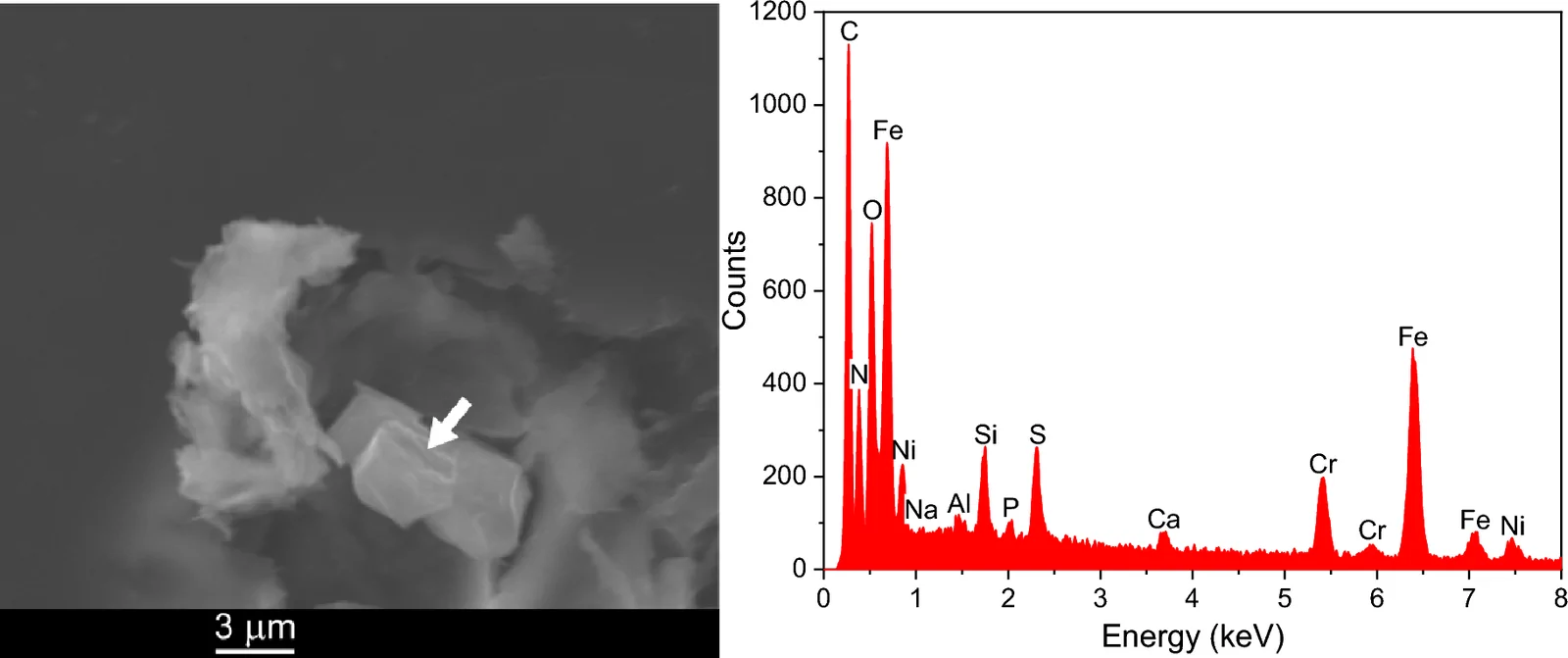
(Left) SEM image showing an irregular iron-rich structure (S3). (Right) The corresponding EDX spectrum of this structure indicates the presence of C, N, O, Na, Mg, Al, Si, P, S, Ca, Cr, Fe and Ni

**Fig. 6:**
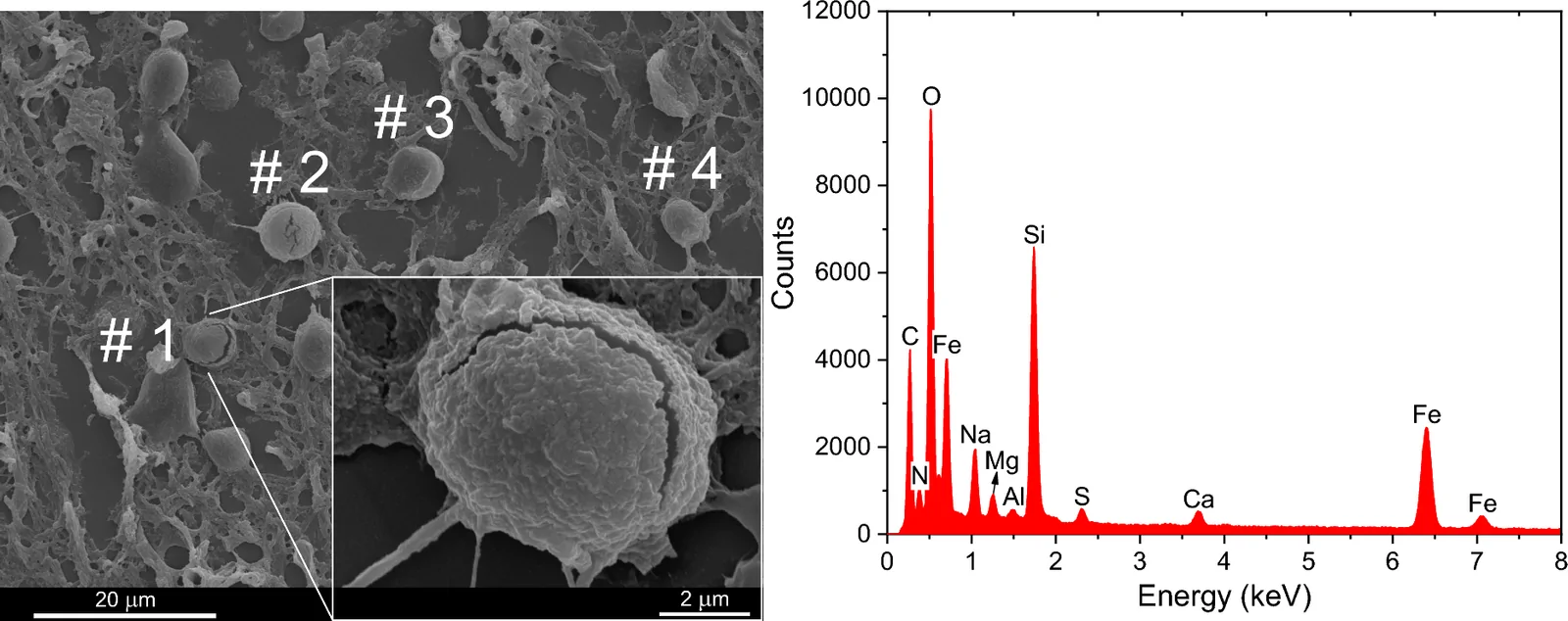
(Left) SEM image of spherical structures (S6). (Right) The corresponding EDX spectrum of the spherical structure #1 indicates the presence of C, N, O, Na, Mg, Al, Si, S, Ca, and Fe

SEM examination of iron-rich particles revealed rectangular, irregular and spherical iron-rich inclusions (Fig. 3–6). Some particles presented as geometrically regular rectangular shapes approximately 5 µm in size (Fig. 3), while others were more extended and irregularly shaped aggregates (Fig. 4 and 5). Elemental analysis of these structures confirmed a multi-elemental composition. The rectangular particle shown in Fig. 3 contained C, N, O, Al, Si, P, S, Ca, and Fe with the highest concentration at the centre of the particle. The irregular structures in Fig. 4 and 5 revealed similar, more complex composition including C, N, O, Na, Al, Si, P, S, Ca, Cr, Fe, and Ni. Silicon comes from glass substrate.

SEM examination of iron-rich particles with sharp bands in the Raman spectra revealed spherical shapes (Fig. 6). The size of all spherical structures was around 6 µm with similar chemical composition containing C, N, O, Na, Mg, Al, Si, S, Ca, and Fe. Silicon comes from the glass substrate.

To distinguish substrate-derived signals from those originating in the biological specimens, an EDX spectrum was acquired from an unused glass slide under identical analytical conditions. The resulting spectrum demonstrated the presence of O, Na, Mg, Si, and Ca, corresponding to the elemental composition of the glass substrate (Kopáni et al. 2025). Consequently, signals associated with these elements in tissue-derived spectra were evaluated in the context of the substrate background and, where appropriate, attributed to the supporting glass slide rather than the tissue itself. This correction was considered in the interpretation of the spectra shown in Figs. 3–6, thereby improving the accuracy of elemental identification within the analysed biological samples.

### Transmission electron microscopy (TEM) and selected area electron diffraction (SAED)

**Fig. 7:**
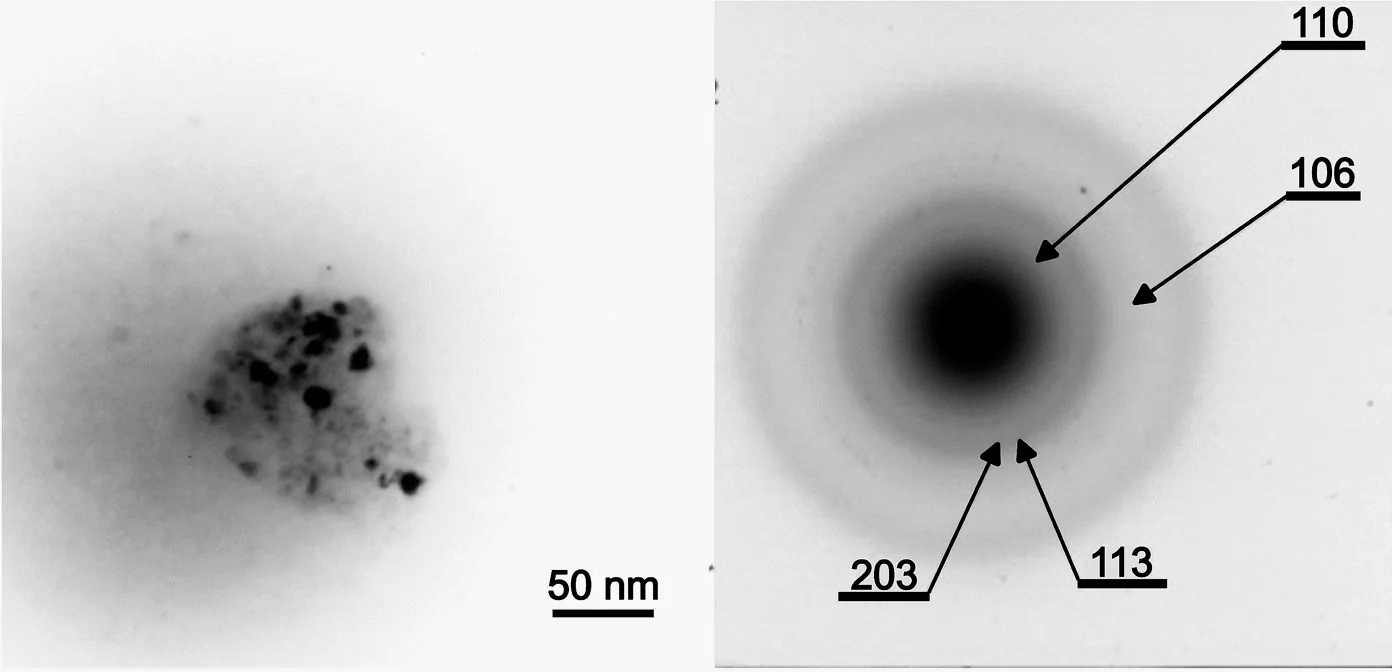
(Left) Dark-field TEM image of an aggregate of nanometre-sized particles from the human *globus pallidus* (S2). (Right) The corresponding SAED pattern exhibits diffuse rings characteristic of nanocrystalline ferrihydrite (ICDD: 29-0712), a likely precursor phase

**Fig. 8:**
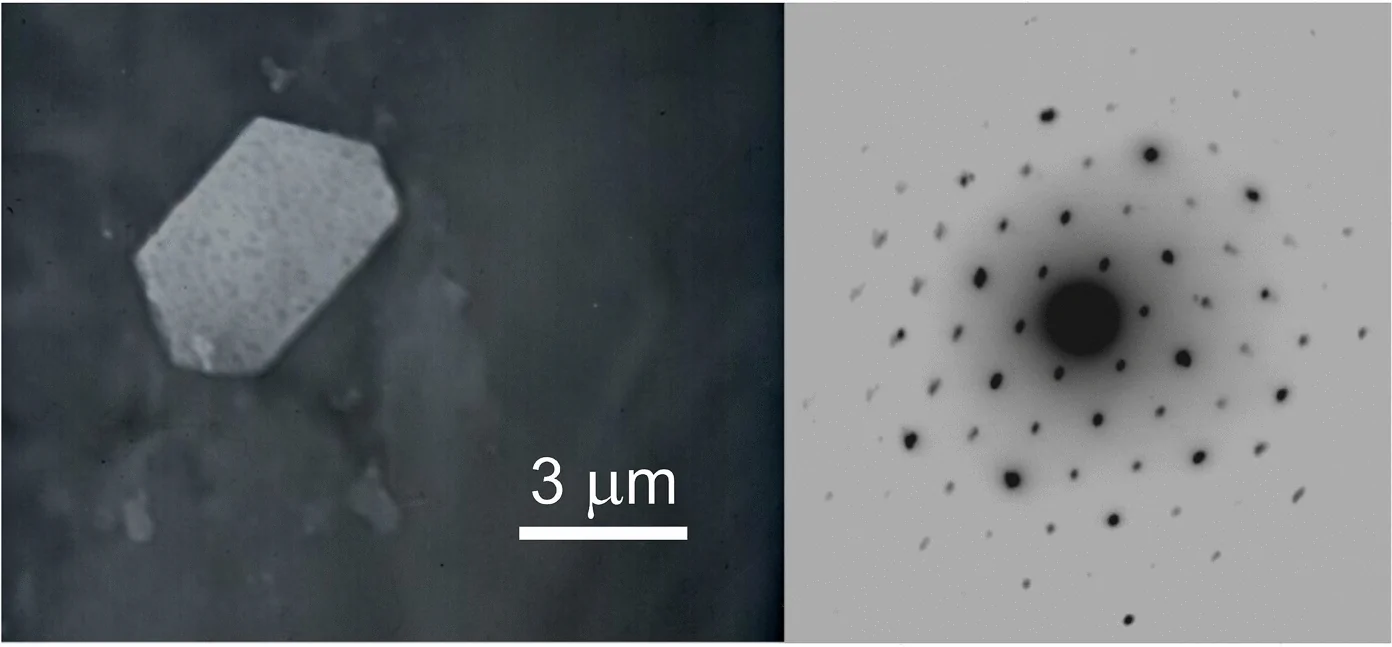
(Left) Dark-field TEM image of a well-formed, hexagonal micrometre-sized particle (S5). (Right) The corresponding single-crystal SAED pattern shows a regular array of diffraction spots indexed to hematite (α–Fe_2_O_3_, ICDD: 33-0664), confirming its high crystallinity

**Fig. 9:**
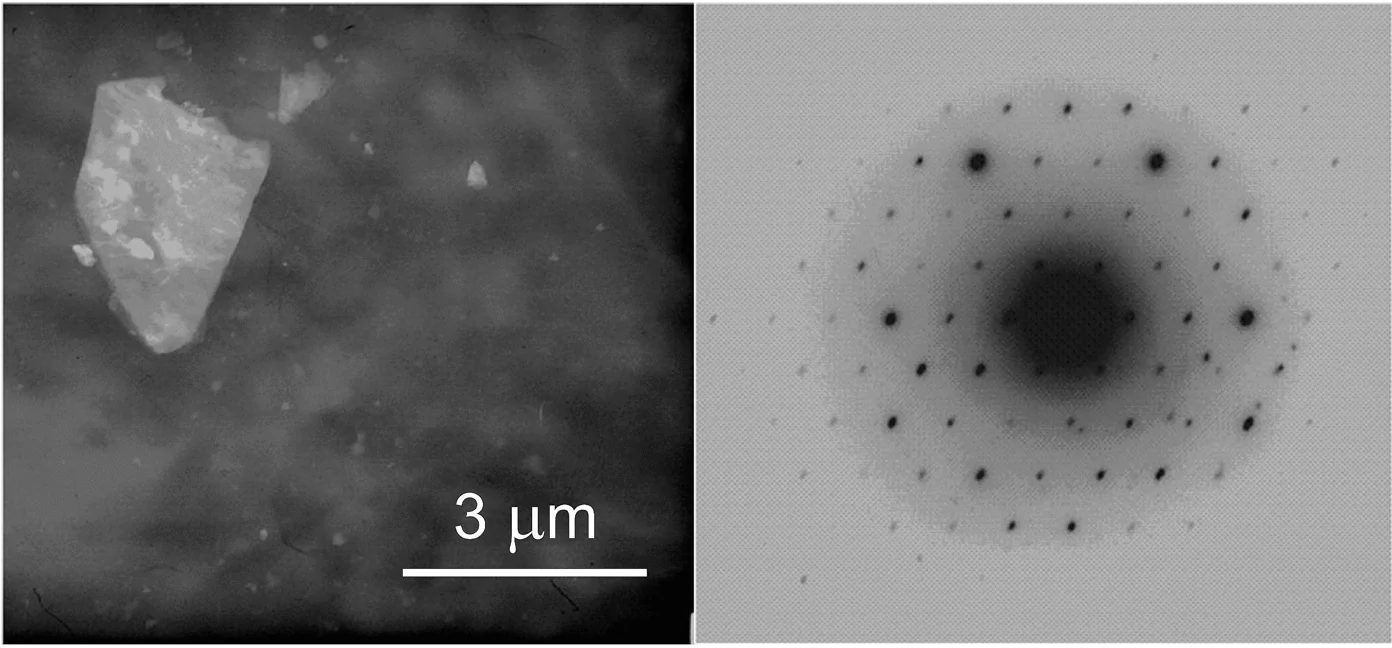
(Left) Dark-field TEM image of a larger, irregularly shaped (surface-roughened) micrometre-sized particle (S4). (Right) The corresponding SAED pattern is consistent with that of crystalline hematite (α–Fe_2_O_3_)

To investigate the crystallinity and ultrastructure of the iron deposits, TEM and SAED analyses were conducted. These investigations revealed two distinct types of iron-containing structures. Firstly, occasional micrometre-scale aggregates were observed, composed of nanometre-sized, oval, electron-dense particles resembling ferritin cores. TEM images in dark field showed that these clusters are made up of nanocrystalline particles ranging in size from about 5 nm to 20 nm. The corresponding SAED pattern produced diffuse diffraction rings characteristic of a nanocrystalline material, which were indexed to ferrihydrite (Figs. 7, 8, 9).

**Fig. 10:**
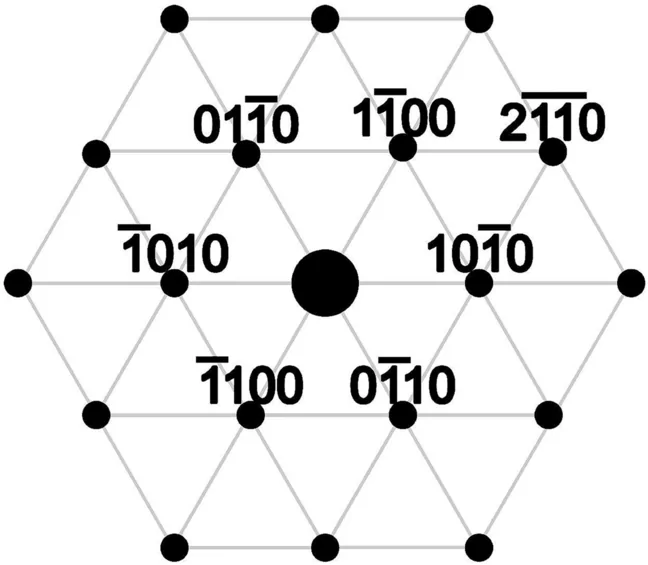
Indexed SAED pattern of the hematite particle in Fig. 8, viewed along the [001] zone axis (α‑Fe_2_O_3_, ICDD: 33‑0664)

Secondly, and in stark contrast, SAED performed on larger, individual microparticles revealed the presence of a well-crystalline material. The diffraction patterns consisted of sharp, distinct spots forming a regular lattice. These patterns were unambiguously indexed to a hexagonal structure with lattice parameters a = 0.503 nm and c = 1.375 nm, corresponding to hematite (α–Fe_2_O_3_). The single-crystal SAED patterns were indexed and the zone axis was determined to confirm the crystallographic orientation of the particles. For the well-formed hexagonal crystal, the regular array of reflections was consistent with hematite viewed along the [001] zone axis (Fig. 10). This indexing further supports the assignment of the microscale particles to crystalline α‑Fe_2_O_3_ rather than to a poorly ordered precursor phase. Particles with this crystalline structure were identified in all six samples investigated by TEM. The morphology of these hematite particles varied. Some particles were well-formed with a distinct hexagonal shape (Fig. 8), while others were larger, surface-roughened, more irregular in shape (Fig. 9). The sizes of these crystalline particles ranged from approximately 1 µm up to 6 µm. On the surface of some particles, small deposits of unknown origin were also observed.

## Discussion

The relationship between iron accumulation in the brain and neurodegeneration is not fully understood. In some rare genetic disorders, such as Neurodegeneration with Brain Iron Accumulation, iron accumulation is considered a direct contributor to neuronal damage. However, in more common neurodegenerative diseases, including Parkinson's and Alzheimer's disease, iron accumulation is often thought to occur as a result of underlying pathological processes. Once excess iron accumulates, it can promote oxidative stress and further damage neurons, creating a vicious cycle in which neurodegeneration leads to iron accumulation, and iron accumulation accelerates neurodegeneration. Therefore, iron is generally viewed not only as a consequence of disease but also as a factor that contributes to its progression. Although increased brain iron has also been associated with normal ageing and cognitive decline, its exact role in these processes remains unclear.

Raman spectroscopy was used to analyse the iron-rich areas in the *globus pallidus* that had been identified via light microscopy after Perls’ histochemical staining. The Raman bands in the low frequency region provide specific insights into the mineralogy of the iron deposits. The vibrations in the 268–278 cm^−1^ range are characteristic of Fe–O bonds in iron oxide lattices, consistent with magnetite (Fe_3_O_4_), hematite (α-Fe_2_O_3_) and/or goethite (α-FeOOH) (de Faria et al. 1997; Shebanova and Lazor 2003). The peak at ~ 526 cm^−1^ can be assigned to magnetite; its shift from the typical value of ~ 540 cm^−1^ may be due to the nanocrystalline nature of the particles, lattice strain, interactions with the surrounding biological matrix or the presence of ions of chemical elements (Slavov et al. 2010; Liu et al. 2013). The band at ~ 603 cm^−1^ is indicative of hematite, which is known to shift to lower frequencies in nanocrystalline form when excited with a red laser (de Faria et al. 1997). The broad feature at 1259–1349 cm^−1^ likely corresponds to the two-magnon scattering of hematite, often seen around 1320 cm^−1^, which is a definitive marker for α-Fe_2_O_3_ (de Faria et al. 1997; Shebanova and Lazor 2003). The presence of maghemite (γ-Fe_2_O_3_) is also possible, as its bands (~ 500 cm^−1^, ~ 700 cm^−1^) can overlap with other signals. Based on assignments (Table 2), these spectral features indicate the presence of magnetite, maghemite, and hematite, as well as ferritin-like structures.

In cells and tissues, iron accumulates mainly in the form of ferritin and hemosiderin. Ferritin is an iron-containing spherical protein approximately 12 nm in size, consisting of a protein shell and a core approximately 6 nm in size composed of iron oxides and hydroxides similar to ferrihydrite, hematite, maghemite, or magnetite (Quintana et al. 2004). Hemosiderin is a storage intracellular pigment containing iron, formed from the breakdown of haemoglobin and partial degradation of ferritin, typically found within macrophages in tissues following haemorrhage or in conditions of systemic iron overload. Unlike ferritin, it does not have a precisely defined structure. The storage of iron in the form of ferritin and hemosiderin serves to safely store iron and protect cells from oxidative stress. Hemosiderin is an insoluble deposit in the form of hydrated ferric oxide aggregates, consisting of proteins, lipids, and remnants of organic compounds (Toyoshima et al. 2023).

The presence of both organic materials and iron oxyhydroxides was confirmed by results from Raman spectroscopy in the high frequency region. The sharper peaks at higher wavenumbers (1298 cm^−1^, 1448 cm^−1^) are indicative of CH_2_/CH_3_ deformations from lipids and proteins, while signals around 1206 cm^−1^ (Amide III) and 1532 cm^−1^ (Amide II) confirm the presence of a proteinaceous matrix (Rygula et al. 2013; Hartmann et al. 2020). The band near 748 cm^−1^, tentatively attributed to (poly)saccharides, may additionally receive a contribution from resonance-enhanced haem/porphyrin modes under 633 nm excitation, which are common in iron- and blood-rich tissue. SEM and EDX analysis revealed spherical structures in the areas with sharper Raman bands. Organic materials cover the iron to prevent the formation of ROS, which can damage the cells and tissue. The spherical shapes showed a similar size of around 6 µm with similar chemical composition. The *globus pallidus* is rich in non-heme iron, which reflects its high metabolic rate. The iron is stored, processed and released through a tightly controlled mechanism (Singh et al. 2014). The observation that iron-containing particles in the *globus pallidus* are coated with organic material and exhibit uniform size and chemical composition suggests a biologically controlled mineralisation. Such homogeneity indicates that the particles likely formed within a stable biochemical microenvironment, where constant pH, redox potential, and specific organic ligands (e.g., carboxyl, hydroxyl, or phenolic groups) control the oxidation of Fe^2+^ and the nucleation of iron oxyhydroxides. The organic coating acts as a template that limits particle growth and maintains their stability. This uniformity therefore reflects both thermodynamic equilibrium and the biological control of iron storage within neural tissue. The two deposit types also differed spectroscopically. The irregular deposits (Fig. 2 left) gave weaker, broader and less-defined bands dominated by the anhydrous, more thermodynamically stable oxides hematite and magnetite. In contrast, the spherical, organic-rich structures (Fig. 2 right), besides their strong organic bands, showed additional weak features tentatively assignable to hydrated or poorly-ordered iron oxyhydroxides–ferrihydrite and, less certainly, lepidocrocite and goethite–together with maghemite. The coexistence of these immature, hydrated phases within the organically templated spherical structures and of well-crystallised hematite and magnetite in the irregular deposits suggests that iron mineralisation in the *globus pallidus* spans a range of maturation states, only part of which appears to be tightly biologically controlled. The sharp band near 748 cm^−1^ in the spherical structures further suggests a (poly)saccharide and/or haem contribution, consistent with these structures representing glycosylated extracellular-matrix components (e.g. proteoglycan-rich assemblies) or lysosomal/lipofuscin-derived material. Their occurrence most likely reflects normal structural features rather than a distinct pathological entity, although definitive molecular identification would require further work. Overall, the Raman-based speciation was in partial agreement with the SAED results: where SAED identified crystalline phases (nanocrystalline ferrihydrite and crystalline hematite), the corresponding Raman features were consistent with these phases, whereas Raman additionally reflected amorphous and poorly ordered contributions not captured by diffraction.

TEM microscopy revealed the presence of ferrihydrite aggregates. The aggregates of ferrihydrite are probably the accumulation of ferritin, the physiological form of iron in the tissue. Individual ferritin cores and their characteristic spacing were not resolved at the resolution of the instrument used (JEOL 840B). The interpretation of these aggregates as accumulated, partly degraded ferritin therefore remains inferential. Ferritin plays a key role in iron storage and protects cells from metal-induced toxicity by sequestering excess metal ions. The incorporation of other metals into the ferritin core can modify its chemical composition, oxidation state, crystallinity, and magnetic and electric properties, potentially affecting its spectroscopic and physicochemical characteristics. Aggregation can lead to the transformation of ferrihydrite into goethite or hematite, which are more stable minerals with lower reactivity. From the TEM examination of the size of ferrihydrite crystals in aggregates, we cannot rule out a transformation to other mineral phases. Aggregates have a smaller specific surface area, which leads to a lower ability to bind contaminants, which is reflected in their toxic effects. Clustering of ferritin in the brain is a process primarily associated with neurodegenerative diseases, which is related to excess iron, oxidative stress, and genetic mutations. Among the most well-known genetic causes are neuroferritinopathy, caused by mutations in the gene for the ferritin light chain (FTL), which alter its structure and lead to the formation of toxic iron aggregates (Levi and Rovida 2015), and aceruloplasminemia, where impaired iron transport causes its accumulation in the brain (Vroegindeweij et al. 2021). In addition to genetic factors, ferritin clustering can also arise due to iron metabolism disorders and increased oxidative stress, which damages nerve cells. Studies show that such changes in ferritin structure and levels may contribute to the development of dementia, Parkinson’s disease, and other neurodegenerative disorders, highlighting the key role of iron in brain health.

### Biomineralisation

The coexistence of multiple iron oxide phases suggests a complex and dynamic biomineralisation process. During this process occurring in the *globus pallidus* tissue, physiological forms of iron oxides can be transformed into other forms and/or they are the results of exogenous pollution. Several studies have confirmed the presence of (nano)particles of magnetite in the human brain (Kirschvink et al. 1992; Gieré 2016) as a result of environmental pollution (Maher et al. 2016) or as a result of transformation from other iron oxides (Van de Walle et al. 2019; Tamura et al. 2020). Magnetite can form in both lipid and protein/carbohydrate environments, and can sometimes be derived from amorphous or semicrystalline ferrihydrite-like material (ferritin). Ferritin itself has been proposed as a precursor for magnetite mineralisation in the brain (Dobson 2001; Van de Walle et al. 2019). The observed phases may reflect ongoing interconversion between less stable iron oxyhydroxides and more thermodynamically stable oxides, influenced by local factors such as pH, redox potential, and the presence of organic molecules (Markovski et al. 2017).

The coexistence of ferrihydrite aggregates (the likely form of iron in ferritin cores) and large, crystalline hematite particles provides strong evidence for a specific biomineralisation pathway. Ferritin nanoparticles in human tissues and cells can aggregate into larger structures (Narayanan et al. 2019). These aggregates of ferrihydrite then serve as a precursor for the formation of crystalline hematite. Ferrihydrite is a metastable phase that can transform into more thermodynamically stable iron oxides such as hematite and goethite (Schwertmann and Murad 1983). Based on the findings, a pathway is proposed whereby this transformation occurs via two competitive mechanisms:

- Solid-state transformation: An internal dehydration and subsequent atomic rearrangement within the ferrihydrite aggregates, resulting in the nucleation and growth of hematite crystals.
- Dissolution-recrystallisation: The dissolution of ferrihydrite into an aqueous solution, followed by the precipitation and crystallisation of goethite.

Vu et al. demonstrated in an in vitro study that hematite can form from ferrihydrite through an aggregation–dehydration–rearrangement process, with goethite acting as an intermediate phase in the presence of water (Vu et al. 2008). The transformation pathways of ferrihydrite and the formation of hematite have also been reported by several other authors (Schwertmann and Murad 1983; Schwertmann et al. 2000; Schwaminger et al. 2017).

Hematite has been identified as redox-active in Fenton-type reactions. Its activity towards the generation of reactive oxygen species (ROS) depends on several factors, including its specific surface area and crystallinity (Prucek et al. 2009), isomorphic substitution of Fe by other cations (Magalhães et al. 2007), pH (Jung et al. 2009), temperature, and particle size (Gordon and Marsh 2009). However, the redox activity of hematite is generally lower than that of other iron oxides, such as magnetite and ferrihydrite (Huang et al. 2001; Lee et al. 2006). Prucek et al. reported that more crystalline hematite exhibits lower catalytic activity than its less-crystalline counterpart, highlighting the importance of structural order in controlling surface reactivity (Prucek et al. 2009). The toxicity of hematite particles is also influenced by particle size. Nanoscale hematite particles have been shown to exhibit slightly higher cytotoxicity and genotoxicity than their microscale counterparts, likely due to differences in cellular uptake and the activation of distinct toxicological pathways. Maintenance of a stable intracellular environment is particularly critical in biological tissues, especially in the brain, where cells are highly sensitive to chemical and oxidative perturbations. In this context, the relatively low redox activity, low solubility, and limited iron release of hematite may contribute to reduced oxidative stress and help preserve cellular homeostasis.

The brain provides a very stable environment in terms of temperature (~ 37 °C) and pH (~ 7.0–7.4) (Ruffin et al. 2014). At near-neutral pH, the dissolution of ferrihydrite is limited, which hinders the solution-based pathway required for goethite formation (Schwertmann and Murad 1983). This favours the solid-state transformation, leading to the preferential formation of hematite. This is consistent with the experimental findings, where crystalline hematite was abundant, but goethite was not identified. In vitro studies have shown that spontaneous ferrihydrite transformation to hematite can occur at room temperature over several years, which aligns with the hypothesis that the micrometre-sized particles observed in this study are the result of a very slow, long-term process, potentially spanning decades of an individual’s life (Schwertmann et al. 1999). The local biochemical environment is a critical factor in directing this mineralisation pathway. The conversion of ferrihydrite to hematite is promoted in a microenvironment rich in water and ROS, particularly OH^−^ radicals (Cornell and Schwertmann 2003). Since iron itself catalyses the production of ROS via Fenton chemistry, it may create a self-propagating environment that supports hematite formation (Cornell and Schwertmann 2003). Furthermore, the rate of iron supply is crucial; slow, chronic iron accumulation favours the formation of well-ordered phases like hematite, which is consistent with the large, highly crystalline particles observed (Cornell and Schwertmann 2003). Organic compounds such as proteins and polysaccharides can also influence the process (Collier and Messersmith 2001; Pavlovic and Brandao 2003). Macromolecules, particularly acidic glycoproteins and proteoglycans with negatively charged residues, can bind iron ions and act as templates for nucleation. Our previous work has shown the presence of iron within globular glycoconjugate structures in the *globus pallidus* (Kopáni et al. 2014). It is proposed that these glycoconjugates may be part of a biological response to inactivate redox-active iron and may be directly involved in templating hematite formation. The specific functional groups of surrounding organic molecules, such as the thiol group in cysteine, the sulphate and carboxylate groups in chondroitin sulphate, or the hydroxyl groups in dextran, can provide different binding sites, leading to different mineralisation outcomes. For instance, a sulphate/carboxylate microenvironment (chondroitin sulphate) has been shown to favour hematite formation, whereas a hydroxyl-rich environment (dextran) favours ferritin-like structures (Yang et al. 1986). The observed heterogeneity of iron-rich deposits in the *globus pallidus* likely reflects differences in iron oxidation state, mineral maturation, and local microenvironmental conditions. Under physiological conditions, iron is primarily stored as ferrihydrite within ferritin; however, alterations in redox balance may promote its transformation into more crystalline phases such as magnetite, maghemite, and hematite. Interactions with surrounding proteins, lipids, and other elements detected by SEM–EDX may further influence mineral formation and stability. The coexistence of multiple iron phases therefore suggests that iron accumulation in the *globus pallidus* is a dynamic process involving storage, oxidation, and mineral transformation, potentially affecting the redox activity of the tissue.

### Pathophysiological and toxicological implications

The accumulation of these heterogeneous iron oxide deposits in the *globus pallidus* may have pathophysiological relevance; however, the present study was performed on neurologically healthy individuals and did not assess any markers of oxidative stress. In principle, crystalline phases such as magnetite and hematite are redox-active and can catalyse the Fenton reaction, generating hydroxyl radicals that promote oxidative stress under conditions of iron dysregulation (Castelnau et al. 1998; Dixon and Stockwell 2014; Ward et al. 2014). Beyond chemical reactivity, the magnetic properties of magnetite have been proposed to disrupt local magnetic fields and potentially interfere with cellular processes such as ion channel function, although this remains hypothetical (Rosen 1993; Pankhurst et al. 2003; Gorobets et al. 2023). However, the redox behaviour of these phases is not uniform. Hematite, in particular, combines low solubility with comparatively low surface reactivity; moreover, for such a thermodynamically stable, bulk crystalline phase any redox activity is essentially confined to the particle surface, while the interior remains largely unreactive. Once formed, these micrometre-sized particles are therefore likely to be highly persistent yet relatively inert and, rather than constituting an acutely toxic species, may represent a long-term, largely sequestered iron reservoir(Cornell and Schwertmann 2003). The biological significance of such deposits therefore remains uncertain. Their mere presence in healthy tissue does not establish a causal role in neurodegeneration, and elevated regional iron may equally reflect physiological storage, age-related accumulation, or a response to glial activity rather than a primary driver of damage. Whether the chronic presence of these particles contributes to a slow, low-level pro-oxidant burden over decades or is essentially benign cannot be resolved from the present data and requires dedicated assessment of oxidative-stress markers in matched healthy and diseased tissue (Chen et al. 2010; Bhattacharya et al. 2012; Freyria et al. 2012; Gustafsson et al. 2015; Lewis et al. 2016).

### Limitations and future directions

A key limitation in the Raman analysis of iron oxides is the potential for laser-induced photothermal transformation. The 633 nm laser used in this study can be absorbed by iron oxides, causing local heating and potentially inducing the oxidation of magnetite to maghemite and subsequently to hematite (de Faria et al. 1997; Shebanova and Lazor 2003). Consequently, some of the detected hematite may be an artefact of the measurement process rather than a reflection of the sample’s native state. Furthermore, the inherent fluorescence background from biological tissue can obscure weak Raman signals, complicating the precise assignment of bands, especially for amorphous or nanocrystalline iron phases. The nanocrystalline nature of these deposits also leads to peak broadening and shifts, making the distinction between magnetite and maghemite particularly challenging. In addition, because the sections were Perls-stained prior to Raman analysis, a minor influence of the staining procedure on the structure of pre-existing iron-oxide particles cannot be entirely excluded, although the principal Prussian blue Raman bands lie outside the measured range and were not observed as a consistent pattern. With respect to sample preparation, independent SQUID-magnetometry and Mössbauer studies have shown that formalin fixation does not measurably alter the iron-oxide phases of brain tissue, so the fixation step is unlikely to have changed the iron speciation reported here (Chua-anusorn et al. 1997; van der Weerd et al. 2020; Walsh et al. 2021). Finally, the cohort was not age-matched; as brain iron content increases with age, inter-individual differences are expected and were taken into account when interpreting the data. To overcome these limitations and build upon these findings, future research should integrate complementary analytical techniques. Mössbauer spectroscopy would provide definitive information on the oxidation state (Fe^2+^/Fe^3+^ ratio) and magnetic properties of the iron phases, while synchrotron X-ray absorption spectroscopy (XANES/EXAFS) is particularly well suited to resolving iron speciation in situ. Used in conjunction with Raman spectroscopy and TEM/SAED, these complementary techniques would provide a more complete and unambiguous characterisation of iron mineralisation in the brain (Lermyte et al. 2020).

## Conclusion

This study demonstrates that iron deposits in the human *globus pallidus* are chemically and structurally heterogeneous, comprising a complex mixture of magnetite, hematite, maghemite, and ferritin-like structures embedded within an organic matrix. These findings support the hypothesis that brain iron deposits are not static but represent a dynamic collection of oxide phases of variable crystallinity and surface reactivity. Depending on these properties, such phases may constitute a potential reservoir of redox-active iron; however, as the present study examined neurologically healthy tissue and did not assess markers of oxidative stress, their physiological versus pathological significance (and any causal role in the oxidative stress implicated in neurodegenerative diseases) remains to be established.

Our work highlights that Raman spectroscopy, when combined with SEM–EDX, is a powerful multimodal approach for gaining molecular-level insights into the heterogeneity of these deposits in situ. While acknowledging the limitations of the technique, particularly potential laser-induced artefacts, our results provide a valuable characterisation of the chemical nature of iron accumulation in a critical brain region. Future studies integrating complementary methods, such as Mössbauer spectroscopy and TEM, are essential to fully resolve the biomineralisation pathways and conclusively determine the role of specific iron phases in the progression of neurodegenerative diseases.

## Data Availability

Dataset is publicly available in a repository ZENODO at the following URL: 10.5281/zenodo.20024113

## References

[CR1] Chua-anusorn W, Webb J, J. Macey D, et al (1997) The effect of histological processing on the form of iron in iron-loaded human tissues. Biochimica et Biophysica Acta (BBA) - Molecular Basis of Disease 1360:255–261. 10.1016/S0925-4439(97)00009-4 10.1016/s0925-4439(97)00009-4 9197468

[CR2] Dobson J (2001) On the structural form of iron in ferritin cores associated with progressive supranuclear palsy and Alzheimer’s disease. Cell Mol Biol (Noisy-le-grand) 47 Online Pub:OL49–50 11936873

[CR3] Chen L, Xie J, Yancey J, et al (2010) Biocompatibility and Delivery of NGF by Hematite Nanotubes for Differentiation of PC12 Cells. J Nanotechnol Eng Med 1:. 10.1115/1.4002746

[CR4] Ruffin VA, Salameh AI, Boron WF, Parker MD (2014) Intracellular pH regulation by acid-base transporters in mammalian neurons. Front Physiol 5:. 10.3389/fphys.2014.00043 10.3389/fphys.2014.00043 PMC3923155 24592239

[CR5] Bhattacharya K, Hoffmann E, Schins RFP et al (2012) Comparison of micro- and nanoscale Fe+3–containing (hematite) particles for their toxicological properties in human lung cells in vitro. Toxicol Sci 126:173–182. 10.1093/toxsci/kfs014 22262566 10.1093/toxsci/kfs014

[CR6] Castellani RJ, Moreira PI, Perry G, Zhu X (2012) The role of iron as a mediator of oxidative stress in Alzheimer disease. BioFactors 38:133–138. 10.1002/biof.1010 22447715 10.1002/biof.1010

[CR7] Castelnau PA, Garrett RS, Palinski W et al (1998) Abnormal iron deposition associated with lipid peroxidation in transgenic mice expressing interleukin-6 in the brain. J Neuropathol Exp Neurol 57:268–282. 10.1097/00005072-199803000-00008 9600219 10.1097/00005072-199803000-00008

[CR8] Collier JH, Messersmith PB (2001) Phospholipid strategies in biomineralization and biomaterials research. Annu Rev Mater Res 31:237–263. 10.1146/annurev.matsci.31.1.237

[CR9] Collingwood JF, Telling ND (2016) Iron oxides in the human brain. Iron oxides. Wiley, pp 143–176

[CR10] Cornell RM, Schwertmann U (2003) The iron oxides: structure, properties, reactions, occurences and uses. Wiley

[CR11] Cowley JM, Janney DE, Gerkin RC, Buseck PR (2000) The structure of ferritin cores determined by electron nanodiffraction. J Struct Biol 131:210–216. 10.1006/jsbi.2000.4292 11052893 10.1006/jsbi.2000.4292

[CR12] de Faria DLA, Venâncio Silva S, de Oliveira MT (1997) Raman microspectroscopy of some iron oxides and oxyhydroxides. J Raman Spectrosc 28:873–878. 10.1002/(SICI)1097-4555(199711)28:11<873::AID-JRS177>3.0.CO;2-B

[CR13] Dixon SJ, Stockwell BR (2014) The role of iron and reactive oxygen species in cell death. Nat Chem Biol 10:9–17. 10.1038/nchembio.1416 24346035 10.1038/nchembio.1416

[CR14] Dünnwald J, Otto A (1989) An investigation of phase transitions in rust layers using raman spectroscopy. Corros Sci 29:1167–1176. 10.1016/0010-938X(89)90052-8

[CR15] Freyria FS, Bonelli B, Tomatis M et al (2012) Hematite nanoparticles larger than 90 nm show no sign of toxicity in terms of lactate dehydrogenase release, nitric oxide generation, apoptosis, and comet assay in murine alveolar macrophages and human lung epithelial cells. Chem Res Toxicol 25:850–861. 10.1021/tx2004294 22324577 10.1021/tx2004294

[CR16] Gieré R (2016) Magnetite in the human body: biogenic vs. anthropogenic. Proc Natl Acad Sci U S A 113:11986–11987. 10.1073/pnas.1613349113 27729531 10.1073/pnas.1613349113 PMC5087066

[CR17] Goldstein J (2003) Scanning electron microscopy and X-ray microanalysis: third edition. Springer US

[CR18] Gordon TR, Marsh AL (2009) Temperature dependence of the oxidation of 2-chlorophenol by hydrogen peroxide in the presence of goethite. Catal Lett 132:349–354. 10.1007/s10562-009-0125-6

[CR19] Gorobets O, Gorobets S, Sharai I et al (2023) Interaction of magnetic fields with biogenic magnetic nanoparticles on cell membranes: physiological consequences for organisms in health and disease. Bioelectrochemistry (Amsterdam) 151:108390. 10.1016/j.bioelechem.2023.108390 10.1016/j.bioelechem.2023.108390 36746089

[CR20] Guo R, Zhang L, Song D et al (2026) Endogenous iron biomineralization in the mouse spleen of metabolic diseases. Fundam Res 6:1535–1544. 10.1016/j.fmre.2024.07.004 42272485 10.1016/j.fmre.2024.07.004 PMC13247509

[CR21] Gustafsson Å, Bergström U, Ågren L et al (2015) Differential cellular responses in healthy mice and in mice with established airway inflammation when exposed to hematite nanoparticles. Toxicol Appl Pharmacol 288:1–11. 10.1016/j.taap.2015.07.001 26163175 10.1016/j.taap.2015.07.001

[CR22] Hagemeier J, Geurts JJ, Zivadinov R (2012) Brain iron accumulation in aging and neurodegenerative disorders. Expert Rev Neurother 12:1467–1480. 10.1586/ern.12.128 23237353 10.1586/ern.12.128

[CR23] Hartmann C, Elsner M, Niessner R, Ivleva NP (2020) Nondestructive chemical analysis of the iron-containing protein ferritin using Raman microspectroscopy. Appl Spectrosc 74:193–203. 10.1177/0003702818823203 30556406 10.1177/0003702818823203

[CR24] Huang H-H, Lu M-C, Chen J-N (2001) Catalytic decomposition of hydrogen peroxide and 2-chlorophenol with iron oxides. Water Res 35:2291–2299. 10.1016/S0043-1354(00)00496-6 11358310 10.1016/s0043-1354(00)00496-6

[CR25] Huang Z, Lui H, Chen XK et al (2004) Raman spectroscopy of in vivo cutaneous melanin. J Biomed Opt 9:1198. 10.1117/1.1805553 15568940 10.1117/1.1805553

[CR26] Hussain A, Yasar M, Ahmad G et al (2023) Synthesis, characterization, and applications of iron oxide nanoparticles. Int J Health Sci (Qassim) 17:3–10 37416845 PMC10321464

[CR27] Jung YS, Lim WT, Park J, Kim Y (2009) Effect of pH on Fenton and Fenton-like oxidation. Environ Technol 30:183–190. 10.1080/09593330802468848 19278159 10.1080/09593330802468848

[CR28] Ke Y, Qian ZM (2003) Iron misregulation in the brain: a primary cause of neurodegenerative disorders. Lancet Neurol 2:246–253. 10.1016/S1474-4422(03)00353-3 12849213 10.1016/s1474-4422(03)00353-3

[CR29] Kim CW, Pawar AU, Hawari T et al (2022) Defectronics based photoelectrochemical properties of Cu2+ ion doped hematite thin film. Sci Rep 12:20972. 10.1038/s41598-022-20045-6 36470878 10.1038/s41598-022-20045-6 PMC9723142

[CR30] Kirschvink JL, Kobayashi-Kirschvink A, Woodford BJ (1992) Magnetite biomineralization in the human brain. Proc Natl Acad Sci U S A 89:7683–7687. 10.1073/pnas.89.16.7683 1502184 10.1073/pnas.89.16.7683 PMC49775

[CR31] Kopáni M, Kopániová A, Čaplovičová M et al (2014) Iron and its relation to glycoconjugates in human globus pallidus. Bratisl Lek Listy 115:362–366. 10.4149/BLL_2014_071 25023427 10.4149/bll_2014_071

[CR100] Kopáni M, Kosnáč D, Pánik J et al (2025) Assessment of the accumulation of certain metals in human globus pallidus using particle-induced X-ray emission (PIXE), scanning electron microscopy (SEM) and energy-dispersive microanalysis (EDX). Appl Sci 15:9897. 10.3390/app15189897

[CR32] Koralewski M, Balejčíková L, Mitróová Z et al (2018) Morphology and magnetic structure of the ferritin core during iron loading and release by magnetooptical and NMR methods. ACS Appl Mater Interfaces 10:7777–7787. 10.1021/acsami.7b18304 29417811 10.1021/acsami.7b18304

[CR33] Langkammer C, Krebs N, Goessler W et al (2010) Quantitative MR imaging of brain iron: a postmortem validation study. Radiology 257:455–462. 10.1148/radiol.10100495 20843991 10.1148/radiol.10100495

[CR34] Lee S, Oh J, Park Y (2006) Degradation of phenol with fenton-like treatment by using heterogeneous catalyst (modified iron oxide) and hydrogen peroxide. Bull Korean Chem Soc 27:489–494. 10.5012/bkcs.2006.27.4.489

[CR35] Lermyte F, Zhang W-Y, Brooks J et al (2020) Metallic iron in cornflakes. Food Funct 11:2938–2942. 10.1039/C9FO02370D 32211629 10.1039/c9fo02370d

[CR36] Levi S, Rovida E (2015) Neuroferritinopathy: from ferritin structure modification to pathogenetic mechanism. Neurobiol Dis 81:134–143. 10.1016/j.nbd.2015.02.007 25772441 10.1016/j.nbd.2015.02.007 PMC4642653

[CR37] Lewis CS, Torres L, Miyauchi JT et al (2016) Absence of cytotoxicity towards microglia of iron oxide (α-Fe2O3) nanorhombohedra. Toxicol Res (Camb) 5:836–847. 10.1039/C5TX00421G 27274811 10.1039/c5tx00421g PMC4890976

[CR38] Liu H, Chen T, Zou X et al (2013) Effect of Al content on the structure of Al-substituted goethite: a micro-Raman spectroscopic study. J Raman Spectrosc 44:1609–1614. 10.1002/jrs.4376

[CR39] Magalhães F, Pereira MC, Botrel SEC et al (2007) Cr-containing magnetites Fe3−xCrxO4: The role of Cr3+ and Fe2+ on the stability and reactivity towards H2O2 reactions. Appl Catal A Gen 332:115–123. 10.1016/j.apcata.2007.08.002

[CR40] Maher BA, Ahmed IAM, Karloukovski V et al (2016) Magnetite pollution nanoparticles in the human brain. Proc Natl Acad Sci U S A 113:10797–10801. 10.1073/pnas.1605941113 27601646 10.1073/pnas.1605941113 PMC5047173

[CR41] Markovski C, Byrne JM, Lalla E et al (2017) Abiotic versus biotic iron mineral transformation studied by a miniaturized backscattering Mössbauer spectrometer (MIMOS II), X-ray diffraction and Raman spectroscopy. Icarus 296:49–58. 10.1016/j.icarus.2017.05.017

[CR42] Matousek P, Stone N (2016) Development of deep subsurface Raman spectroscopy for medical diagnosis and disease monitoring. Chem Soc Rev 45:1794–1802. 10.1039/C5CS00466G 26455315 10.1039/c5cs00466g

[CR43] Mazzetti L, Thistlethwaite PJ (2002) Raman spectra and thermal transformations of ferrihydrite and schwertmannite. J Raman Spectrosc 33:104–111. 10.1002/jrs.830

[CR44] Meguro R, Asano Y, Odagiri S et al (2007) Nonheme-iron histochemistry for light and electron microscopy: a historical, theoretical and technical review. Arch Histol Cytol 70:1–19. 10.1679/aohc.70.1 17558140 10.1679/aohc.70.1

[CR45] Narayanan S, Shahbazian-Yassar R, Shokuhfar T (2019) Transmission electron microscopy of the iron oxide core in ferritin proteins: current status and future directions. J Phys D Appl Phys 52:453001. 10.1088/1361-6463/ab353b

[CR46] Pankhurst QA, Connolly J, Jones SK, Dobson J (2003) Applications of magnetic nanoparticles in biomedicine. J Phys D Appl Phys 36:R167–R181. 10.1088/0022-3727/36/13/201

[CR47] Pavlovic S, Brandao PRG (2003) Adsorption of starch, amylose, amylopectin and glucose monomer and their effect on the flotation of hematite and quartz. Miner Eng 16:1117–1122. 10.1016/j.mineng.2003.06.011

[CR48] Perez-Gonzalez T, Jimenez-Lopez C, Neal AL et al (2010) Magnetite biomineralization induced by *Shewanella oneidensis*. Geochim Cosmochim Acta 74:967–979. 10.1016/j.gca.2009.10.035

[CR49] Prucek R, Hermanek M, Zbořil R (2009) An effect of iron(III) oxides crystallinity on their catalytic efficiency and applicability in phenol degradation—A competition between homogeneous and heterogeneous catalysis. Appl Catal A Gen 366:325–332. 10.1016/j.apcata.2009.07.019

[CR50] Quintana C, Gutiérrez L (2010) Could a dysfunction of ferritin be a determinant factor in the aetiology of some neurodegenerative diseases? Biochimica Et Biophysica Acta (BBA) - General Subjects 1800:770–782. 10.1016/j.bbagen.2010.04.012 20447447 10.1016/j.bbagen.2010.04.012

[CR51] Quintana C, Cowley JM, Marhic C (2004) Electron nanodiffraction and high-resolution electron microscopy studies of the structure and composition of physiological and pathological ferritin. J Struct Biol 147:166–178. 10.1016/j.jsb.2004.03.001 15193645 10.1016/j.jsb.2004.03.001

[CR52] Ramos P, Santos A, Pinto NR et al (2014) Iron levels in the human brain: a post-mortem study of anatomical region differences and age-related changes. J Trace Elem Med Biol 28:13–17. 10.1016/j.jtemb.2013.08.001 24075790 10.1016/j.jtemb.2013.08.001

[CR53] Rosen AD (1993) A proposed mechanism for the action of strong static magnetic fields on biomembranes. Int J Neurosci 73:115–119. 10.3109/00207459308987217 7510673 10.3109/00207459308987217

[CR54] Rouault TA (2013) Iron metabolism in the CNS: implications for neurodegenerative diseases. Nat Rev Neurosci 14:551–564. 10.1038/nrn3453 23820773 10.1038/nrn3453

[CR55] Rygula A, Majzner K, Marzec KM et al (2013) Raman spectroscopy of proteins: a review. J Raman Spectrosc 44:1061–1076. 10.1002/jrs.4335

[CR56] Schwaminger SP, Bauer D, Fraga-García P et al (2017) Oxidation of magnetite nanoparticles: impact on surface and crystal properties. CrystEngComm 19:246–255. 10.1039/C6CE02421A

[CR57] Schwertmann U, Murad E (1983) Effect of pH on the formation of goethite and hematite from ferrihydrite. Clays Clay Miner 31:277–284. 10.1346/CCMN.1983.0310405

[CR58] Schwertmann U, Friedl J, Stanjek H (1999) From Fe(III) ions to ferrihydrite and then to hematite. J Colloid Interface Sci 209:215–223. 10.1006/jcis.1998.5899 9878155 10.1006/jcis.1998.5899

[CR59] Schwertmann U, Friedl J, Stanjek H, Schulze DG (2000) The effect of Al on Fe oxides. XIX. Formation of Al-substituted hematite from ferrihydrite at 25°C and pH 4 to 7. Clays Clay Miner 48:159–172. 10.1346/CCMN.2000.0480202

[CR60] Shahabi S, Fekrazad R, Johari M et al (2016) FT-Raman spectroscopic characterization of enamel surfaces irradiated with Nd:YAG and Er:YAG lasers. J Dent Res Dent Clin Dent Prospects 10:207–212. 10.15171/joddd.2016.033 28096945 10.15171/joddd.2016.033 PMC5237666

[CR61] Shebanova ON, Lazor P (2003) Raman spectroscopic study of magnetite (FeFe2O4): a new assignment for the vibrational spectrum. J Solid State Chem 174:424–430. 10.1016/S0022-4596(03)00294-9

[CR62] Singh N, Haldar S, Tripathi AK et al (2014) Brain iron homeostasis: from molecular mechanisms to clinical significance and therapeutic opportunities. Antioxid Redox Signal 20:1324–1363. 10.1089/ars.2012.4931 23815406 10.1089/ars.2012.4931 PMC3935772

[CR63] Slavov L, Abrashev MV, Merodiiska T et al (2010) Raman spectroscopy investigation of magnetite nanoparticles in ferrofluids. J Magn Magn Mater 322:1904–1911. 10.1016/j.jmmm.2010.01.005

[CR64] Spencer P, Ye Q, Kamathewatta NJB et al (2021) Chemometrics-assisted Raman spectroscopy characterization of tunable polymer-peptide hybrids for dental tissue repair. Front Mater. 10.3389/fmats.2021.681415 34113623 10.3389/fmats.2021.681415 PMC8186416

[CR65] Svobodová H, Hlinková J, Janega P et al (2019) Deposits of iron oxides in the human globus pallidus. Open Phys 17:291–298. 10.1515/phys-2019-0030

[CR66] Tamura K, Kunoh T, Nakanishi M et al (2020) Preparation and characterization of additional metallic element-containing tubular iron oxides of bacterial origin. ACS Omega 5:27287–27294. 10.1021/acsomega.0c03574 33134691 10.1021/acsomega.0c03574 PMC7594126

[CR67] Thomas M, Jankovic J (2004) Neurodegenerative disease and iron storage in the brain. Curr Opin Neurol 17:437–442. 10.1097/01.wco.0000137534.61244.d1 15247539 10.1097/01.wco.0000137534.61244.d1

[CR68] Toyoshima T, Harada Y, Morikawa T, Shimizu T (2023) Dapsone-induced Heinz-body haemolytic anaemia. BMJ Case Rep 16:e256775. 10.1136/bcr-2023-256775 37802591 10.1136/bcr-2023-256775 PMC10565255

[CR69] Van de Walle A, Plan Sangnier A, Abou-Hassan A et al (2019) Biosynthesis of magnetic nanoparticles from nano-degradation products revealed in human stem cells. Proc Natl Acad Sci U S A 116:4044–4053. 10.1073/pnas.1816792116 30760598 10.1073/pnas.1816792116 PMC6410821

[CR70] van der Weerd L, Lefering A, Webb A et al (2020) Effects of Alzheimer’s disease and formalin fixation on the different mineralised-iron forms in the human brain. Sci Rep 10:16440. 10.1038/s41598-020-73324-5 33020534 10.1038/s41598-020-73324-5 PMC7536241

[CR71] Vroegindeweij LHP, Bossoni L, Boon AJW et al (2021) Quantification of different iron forms in the aceruloplasminemia brain to explore iron-related neurodegeneration. Neuroimage Clin 30:102657. 10.1016/j.nicl.2021.102657 33839643 10.1016/j.nicl.2021.102657 PMC8055714

[CR72] Vu HP, Shaw S, Benning LG (2008) Transformation of ferrihydrite to hematite: an *in situ* investigation on the kinetics and mechanisms. Mineral Mag 72:217–220. 10.1180/minmag.2008.072.1.217

[CR73] Walsh KJ, Shah SV, Wei P et al (2021) Effects of fixatives on histomagnetic evaluation of iron in rodent spleen. J Magn Magn Mater 521:167531. 10.1016/j.jmmm.2020.167531 33343059 10.1016/j.jmmm.2020.167531 PMC7748249

[CR74] Ward RJ, Zucca FA, Duyn JH et al (2014) The role of iron in brain ageing and neurodegenerative disorders. Lancet Neurol 13:1045–1060. 10.1016/S1474-4422(14)70117-6 25231526 10.1016/S1474-4422(14)70117-6 PMC5672917

[CR75] Willans M, Hollings A, Boseley RE et al (2025) The application of X-ray fluorescence microscopy and micro-XANES spectroscopy to study neuro-metallomics. J Inorg Biochem 262:112744. 10.1016/j.jinorgbio.2024.112744 39341704 10.1016/j.jinorgbio.2024.112744

[CR76] Yang C, Bryan AM, Theil EC et al (1986) Structural variations in soluble iron complexes of models for ferritin: an x-ray absorption and mössbauer spectroscopy comparison of horse spleen ferritin to blutal (iron-chondroitin sulfate) and imferon (iron-dextran). J Inorg Biochem 28:393–405. 10.1016/0162-0134(86)80025-3 3102689 10.1016/0162-0134(86)80025-3

[CR77] Zecca L, Youdim MBH, Riederer P et al (2004) Iron, brain ageing and neurodegenerative disorders. Nat Rev Neurosci 5:863–873. 10.1038/nrn1537 15496864 10.1038/nrn1537

